# Devulcanization Technologies for Recycling of Tire-Derived Rubber: A Review

**DOI:** 10.3390/ma13051246

**Published:** 2020-03-10

**Authors:** Erich Markl, Maximilian Lackner

**Affiliations:** University of Applied Sciences FH Technikum Wien, A-1200 Vienna, Austria; erich.markl@technikum-wien.at

**Keywords:** rubber devulcanization, sustainability, recycling, twin screw extruder, feedstock recycling, magic triangle, elastomer

## Abstract

In general, composite materials are difficult to recycle. Tires belong to this class of materials. On top, one of their main constitutents, vulcanized rubber, is as elastomer, which cannot be remolten and hence is particularly challenging to put to a new use. Today, the main end-of-life routes of tires and other rubber products are landfilling, incineration in e.g., cement plants, and grinding to a fine powder, generating huge quantities and indicating a lack of sustainable recycling of this valuable material. True feedstock recycling is not feasible for complex mixtures such as tires, but devulcanization can be done to reactivate the cross-linked polymer for material recycling in novel rubber products. Devulcanization, i.e., the breaking up of sulfur bonds by chemical, thermophysical, or biological means, is a promising route that has been investigated for more than 50 years. This review article presents an update on the state-of-the art in rubber devulcanization. The article addresses established devulcanization technologies and novel processes described in the scientific and patent literatures. On the one hand, tires have become high-tech products, where the simultaneous improvement of wet traction, rolling resistance, and abrasion resistance (the so-called “magic triangle”) is hard to achieve. On the other hand, recycling and sustainable end-of-life uses are becoming more and more important. It is expected that the public discussion of environmental impacts of thermoplastics will soon spill over to thermosets and elastomers. Therefore, the industry needs to develop and market solutions proactively. Every year, approximately 40 million tons of tires are discarded. Through the devulcanization of end-of-life tires (ELT), it is possible to produce new raw materials with good mechanical properties and a superior environmental footprint over virgin products. The devulcanization process has become an interesting technology that is able to support the circular economy concept.

## 1. Introduction

With increasing global populations and welfare, consumption has been surging. Polymers—thermoplastics, thermosets, and elastomers—have shown significant growth over more than six decades from the 1950s onwards, with thermoplastics being by far the largest group. In 2018, the production volume has approached 350 million tons [1]. The steady, historic growth rate of 6% per year is expected to flatten considerably in the coming years due to a pressure toward recycling plastics materials. Plastics Europe and other associations have shifted their focus of communication from job and value creation of the industry toward recycling and littering prevention; the circular economy, sustainability, microplastics pollution, and prevention have become common concerns, which the industry is starting to address seriously. Despite the huge efforts put into the recycling of thermoplastics, the achievements have been rather disappointing, apart from selected successful recycling schemes such as PET (polyethylene terephthalate) with bottles of carbonated soft drinks. “Thermal recycling” sounds nice; however, it should only be considered as the last step of a cascaded use, since the incineration to recapture energy is adding little value. Composite materials such as GFRP and CFRP (glass fiber-reinforced plastics and carbon fiber-reinforced plastics) make recycling extremely difficult as well as the variety of applications of plastics and various contaminations such as foodstuffs. PET bottles can be collected and recycled efficiently and effectively, because carbonated soft drinks and bottled water are put almost exclusively into PET containers. Packaging film, on the other hand, is often a multilayer material that is used particularly for perishable food, where recycling becomes virtually impossible. The low value of plastics, compared to other materials, makes recycling challenging, too. Plastics Europe, in one of their recent reports, claims that within the EU28 (Belgium, Bulgaria, Czech Republic, Denmark, Germany, Estonia, Ireland, Greece, Spain, France, Croatia, Italy, Cyprus, Latvia, Lithuania, Luxembourg, Hungary, Malta, Netherlands, Austria, Poland, Portugal, Romania, Slovenia, Slovakia, Finland, Sweden, United Kingdom), Norway, and Switzerland, in 2016, 31.1% of the 27.1 million tons of post-consumer waste collected plastics were recycled, of which 63% were inside the EU, and another 41.6% were sent to energy recovery, with 27.3% remaining for landfilling (the landfilling ban in the EU came into force in 1999) [2]. These numbers are misleading, because the total demand was in excess of 50 million tons, and the absolute recycling rates, although they are increasing from year to year driven by landfill restrictions for organic materials, are disappointingly low. Recycled thermoplastics go different routes. Production scrap is recycled most easily; typically, 10%–15% of own material (e.g., sprues in injection molding) can be shredded and added without quality issues. Post-consumer recycled plastics can go into products of lower mechanical properties. Prices of recycled polyolefins, due to consumers’ demand for “green” products, have increased sharply in the last years. Another promising route are bioplastics, which can either be based on renewable raw materials and/or be biodegradable. Currently, their market share is on the order of 1%–2% of global plastics consumption. For polymers (thermoplastics), there are typically two recycling methods: mechanical and thermal (the latter being incineration for energy recovery). Garforth et al. have defined feedstock recycling as a process that "aims to convert waste polymer into original monomers or other valuable chemicals" [3]. Synonyms for feedstock recycling are chemical recycling or tertiary recycling. According to Aguado et al. [4], one can distinguish between three main approaches in feedstock recycling: depolymerization, partial oxidation, and cracking (thermal, catalytic, and hydrocracking). Kaminsky at al. have studied the feedstock recycling of synthetic and natural rubber by pyrolysis in a fluidized bed [5]. The main issue was that the original monomers are hard to obtain and that rather a mix of different molecules results. Some authors even understand the production of low-value products such as carbon black as feedstock recycling.

In the case of tires, which are a complex product made from completely different raw materials such as steel, cord, natural and synthetic rubber, additives, etc., full feedstock recycling will not be feasible, i.e., obtaining the original constituents or monomers. 

“Feedstock recycling” and “devulcanization” are two terms that are rather not to be used interchangeably, since the ambition is different. The expressions “depolymerization” or “molecular rearrangement” hit the meaning of devulcanization better.

**Figure 1:**
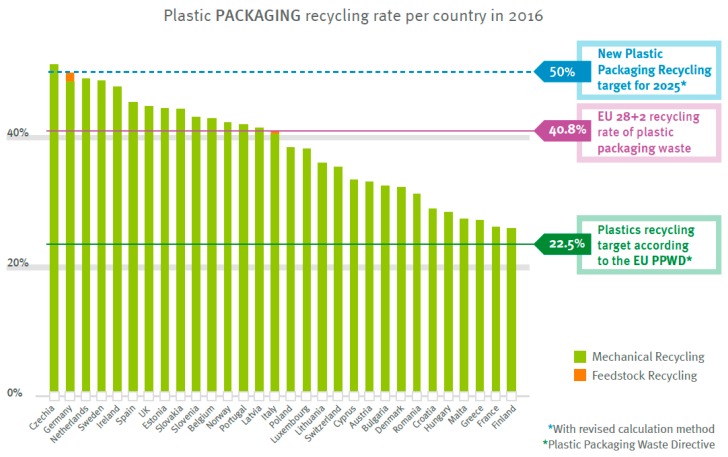
Feedstock recycling of thermoplastics packaging is still in its infancy. Reproduced with permission from [1].

True feedstock recycling can be considered the “holy grail” of plastics recycling in that the monomers are obtained from collected scrap, and then, they are captured and reused. However, this route has not yet been developed sufficiently, and many approaches are still at a low technology readiness level. Figure 1 shows the extent of feedstock recycling for thermoplastics packaging materials (more recent data were not given in the 2018 report).

Another project in this direction is the Austrian mineral oil company OMV’s ReOil project, where 100 kg/h of plastics waste can be converted into a type of synthetic crude [6].

For thermosets, recycling as for thermoplastics is not feasible, because the polymer chains have been converted into a rigid network that cannot be dissolved or molten anymore. There are some attempts to e.g., burn off the polymer matrix to recycle fibers from composite materials, which in an energy-efficient process can make sense for high-value materials such as carbon fibers. 

For elastomers, recycling options are strongly limited, too, because the polymer is also a network. Elastomers cannot be molten nor be dissolved. One of the huge volume applications of elastomers is tires, in which natural rubber is used next to a mix of synthetic rubbers. By vulcanization or curing, the properties of the natural rubber compounds are finalized (a low sulfur content on the order of 2% yields soft rubber, whereas more sulfur addition gives hard rubber). However, the biodegradability of the raw materials (mostly latex) is thereby lost. Tires are produced (and discarded) on the order of 40 million tons per year on a global basis, and they have become a huge environmental concern.

Whereas waste tire dumps are visible to the public and are of general concern, end-of-life options for tires include incineration in cement plants and grinding them to a fine powder for addition into asphalt or concrete, which are rarely discussed in the general public. The attrition of tires on the roads which leads to microplastics formation is studied and discussed even less [7], although it bears a strong environmental impact. 

In the case of tires and rubber in general, feedstock recycling would be a very beneficial approach. For more than five decades, the devulcanization of rubber has been studied. Different technologies have been developed, and some of them have already made it to the market. This review article provides an update on the state-of-the-art in rubber devulcanization with an outlook on potential future developments.

## 2. Materials and Methods 

### 2.1. Rubber Vulcanization

By vulcanization, as invented by Goodyear, sulfur can form bonds between unsaturated polymer chains found in latex to yield natural rubber. The process is also used for synthetic rubber. Accelerators can be added in the process, which is carried out at elevated temperatures. Accelerated sulfur vulcanizations are classified into three different types such as conventional (CV), semi-efficient (semi-EV), and efficient vulcanization (EV) depending on accelerator/sulfur ratio (A/S) between 0.1 and 12 [8].

Common vulcanization accelerators are MBT, TBBS, TMTD, DPG, and CBS; for definitions, see Table 1 [8].

Vulcanization gives the properties to natural or synthetic rubber; it renders the material into an elastomer.

### 2.2. Waste Tires

Tires are used on all sorts of vehicles. After several years, they need to be replaced, because their profiles have become worn out, and/or they have become brittle.

Retreading is done for truck tires, while passenger car tires are single-use items. End-of-life-tires (ELTs) can be mainly recovered through two routes: the recovery of material and the recovery of energy [9]. The calorific value of ELT is close to that of coal, and they are often used in paper mills and cement works. By pyrolysis, oils can be made [10], as deployed e.g., in rural China on a scale of 2 million tons per year [11], leading to substantial emissions. Another possible outlet is oilspill remediation [12]. Material recovery [13] requires the granulation/grinding of ELTs. The grinding is reviewed in [14] by Asaro et al. One can distinguish between ambient, wet, and cryogenic grinding. Most technologies for tire recycling involve the separation of metallic and textile (cord) materials and a grinding process leading to a significant reduction of the tire dimensions. During the grinding process, which typically yields granulate of a few mm or below, the temperature can be lower than the glass transition temperature (i.e., cryogenic grinding) of the polymers in the tires or close to room temperature. The resulting powder can be used as a filler e.g., in new tire compounds but with only a little amount added at a time. The compatibility between the new rubber compound and ELTs or their powder can be increased. Therefore, the ELTs must be devulcanized by breaking the three-dimensional cross-linking network, or they must be modified on the surface [9].

### 2.3. Rubber Devulcanization

Elastomers such as rubber are cross-linked, which prevents simple recycling, as it can be applied to thermoplastics [15,16]. The devulcanization process aims at selectively cleaving the C-S bonds while leaving the C-C bonds intact. The devulcanization of waste rubber applies energy to the material in order to break up, totally or partially, the three-dimensional network formed during vulcanization [17]. Selectivity is difficult to achieve, since the energies that are needed to break the S-S and C-S bonds (227 and 273 kJ/mol, respectively) are rather close to the energy required to break the C-C bonds (348 kJ/mol) [18].

The higher the selectivity of the devulcanization process, the better will be the mechanical properties of the material. Horikx [19] has developed a tool for investigating the mechanism of network breakdown in a vulcanized rubber network. According to this theory, the rate of increase of the soluble (sol) fraction of the rubber as a function of the measured cross-link density of the remaining insoluble (gel) fraction is different for cleavage of carbon–sulfur and carbon–carbon bonds. Thus, sol fraction and cross-linking density measurements of devulcanized rubber samples yield an indication of the dominant mechanism of network breakdown according to Edards et al. [20].

**Figure 2:**
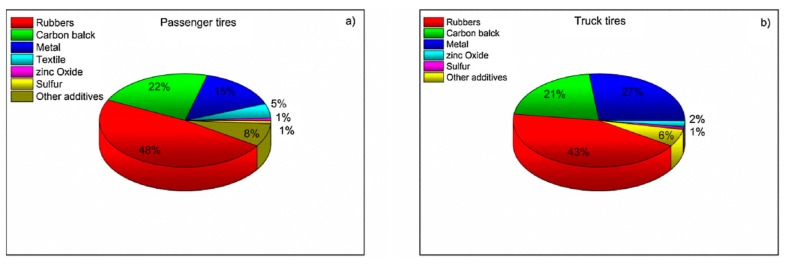
Tire composition for passenger cars (left) and trucks (right). Reproduced with permission from: [14].

It is estimated that 70% of global rubber production goes into tires, which consist of up to 60% of natural and synthetic rubber [14]. Therefore, waste tires are considered the main resource for rubber reclaiming and recycling; comparisons are shown in Figure 2.

Truck tires contain more natural rubber than tires for passenger cars, because they are subjected to more mechanical stress, which natural rubber sustains better [14].

Truck tires typically contain natural rubber (NR) and synthetic rubber (butadiene rubber, BR and styrene–butadiene rubber, SBR) [9,21].

The basic composition of tire rubber is shown in Table 2 below.

The tire rubber composition as shown in Table 2 strongly affects, or rather determines, the product properties. Tires play a critical role in a vehicle’s safety performance, operating costs, and environmental impact. The industry constantly aims at improving wet traction, rolling resistance, and abrasion resistance, which has become known as the “magic triangle”, since the optimization of any of these parameters typically leads to a worsening of the others. With tires having become high-tech products, the raw materials need to be well-defined with constant properties, which makes it difficult to develop recycling materials, particularly since tires are not uniform in their composition.

Regarding the useful life, there are two types of tires: the reusable tires and the non-reusable tires. The reusable tires are sent to tire retreading companies, providing them a new tread and run into service again. The non-reusable are tires that cannot be retreaded due to an advanced damage, structural deformation, or high degradation. These tires are the starting materials for recycling, according to Asaro et al. [14].

For devulanization, waste rubber tire (WRT) material is typically first processed into ground tire rubber (GTR). While waste tires are often just landfilled or burnt in an ill-controlled manner, GTR can be processed into rubberized asphalt [23,24], bitumen [25], cement [26], concrete, tiles, thermal and acoustic isolation [14], and other products. However, simply mixing untreated GTR into an (elastomeric) matrix greatly decreases its mechanical properties, because the cross-linked rubber particles will show poor interfacial adhesion and dispersion.

To improve these, devulcanization has been researched for more than five decades [27]. 

In the process, monosulfidic, (C-S), di-sulfidic (S-S), and polysulfidic (–S_x_-) bonds in the rubber matrix are cleaved.

It was shown by de Sousa et al. that the final temperature reached by the rubber mixture is the main factor responsible for the success of the process [28]. Too high temperatures are to be avoided to prevent degradation of the main chains.

Thermomechanical [7,18], chemical [29,30,31], ultrasonic-based [32], microwave-assisted [28,33], and biological devulcanization methods [34] have been studied extensively.

Molanorouzi and Mohaved have proposed an irradiation technique for rubber devulcanization [35]. Chen et al. [36] describe supercritical solvent-based devulcanization.

A twin-screw extruder for thermomechanical devulcanization is considered most practical [18], because that type of machinery is commonly used in the polymer industry. In addition, scalability to industrial volume is seen best for extrusion [14].

**Figure 3:**
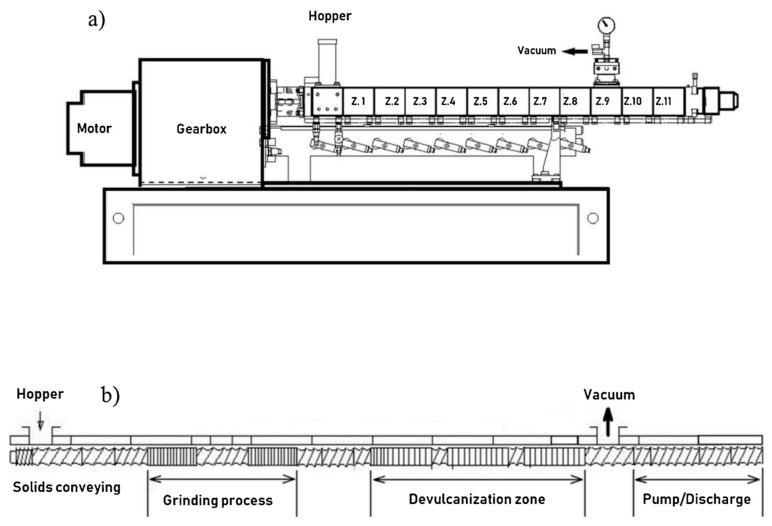
A twin-screw extruder for continuous devulcanization. Reproduced with permission from [18], (**a**) Shows a scheme of the extruder. (**b**) is a typical screw configuration with the different process sections.

Figure 3 shows a typical co-rotating twin-screw extruder setup (a) and a screw configuration for devulcanization (b).

Formela et al. have studied the effect of screw configuration [37].

It was found by Seghar et al. that up to 65 wt % of virgin natural rubber (NR) can be replaced by rubber recycled with devulcanized material [18]. In general, lower temperatures than for vulcanization are deployed to avoid the formation of harmful volatile organic compounds (VOC) and destructive polymer degradation (cleavage of C-C bonds). H_2_S and mercaptanes are toxic compounds, and the resulting fumes need to be captured and controlled. A process temperature of 180–300 °C is often recommended. Seghar et al. [18] have used 80–220 °C. 

**Figure 4:**
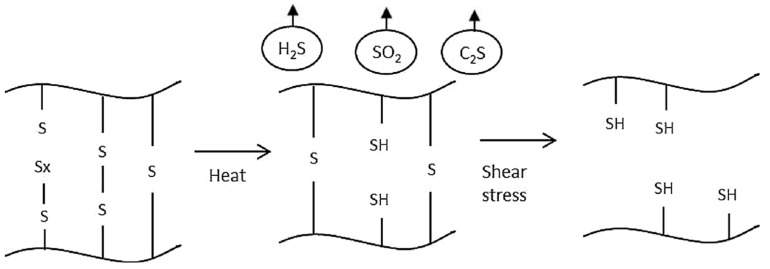
Mechanism for the cross-linking breakage in a thermomechanical devulcanization process. Reproduced with permission from [14].

Figure 4 takes a look at the postulated devulvanization mechanism.

For the thermochemical approach in an extruder, the use of supercritical CO_2_ (scCO₂) has been suggested [14,38]. CO_2_ is chemically inactive, non-toxic, non-flammable, and inexpensive. Its critical point can be reached easily (31.1 °C, 7.38 MPa), and residual scCO₂ in the devulcanized rubber is removed easily.

**Figure 5:**
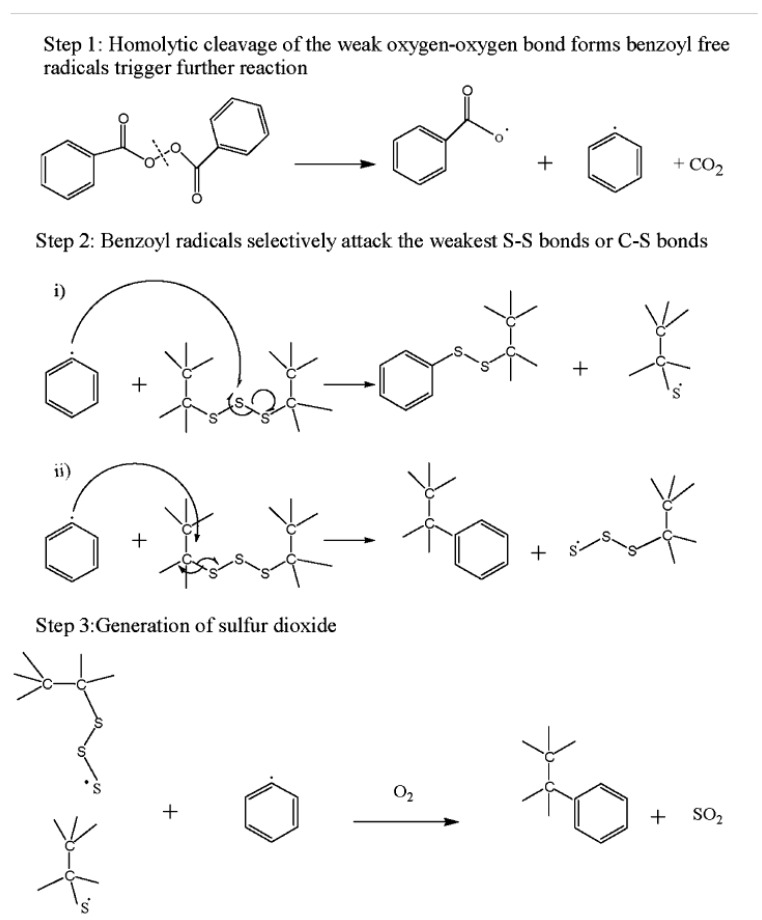
Plausible reaction mechanism of devulcanization. Reproduced with permission from [39].

As a chemical method, the oxidation of sulfur bonds using nitric acid (HNO_3_) and benzoyl-peroxide (C_14_H_10_O_4_) was studied [30,31,39]. Figure 5 takes a look at the mechanism using that agent.

Asaro et al. [14] have suggested diphenyl disulfide (DD) as effective devulcanizing agent. DD was also proposed by other authors such as Kojima et al. [40,41,42,43], Jiang et al. [44], Shi et al. [45], and Mangili et al. [46].

Mangili et al. [9] used ground truck-tire rubber (GTR) for devulcanization in supercritical CO_2_ in the presence of diphenyl disulfide as the devulcanizing agent.

The temperature and pressure were 180 °C and 15 MPa, and the ratio between rubber and DD was 10 wt %.

ScCO_2_ was found to be a good swelling agent, and it exhibits a favorable distribution coefficient for DD [9]. The most limiting factor for this devulcanization process is the amount of unreacted DD in the treated GTR [9].

For ultrasound, 20–50 kHz were proposed by Liang et al. [47].

Concerning devulcanization by microwaves, it was found out by de Sousa et al. that the natural rubber phase of tires, which contains most of the carbon black as opposed to the synthetic rubber phase, can be degraded more by microwaves [28].

An alternative approach has been the use of ionic liquids as studied by Seghar et al. [48]. To improve the devulcanization efficiency, Saputra et al. [49] have tested deep eutectic solvents (DES) in thermochemical–ultrasonic devulcanization of GTR. As DES, ChCl:urea, ChCl:ZnCl_2_, and ZnCl_2_:urea were used, with ChCl being choline chloride.

Thiobisphenols, e.g., 4,40-dithiobis(2,6-di-t-butylphenol), were also studied for thermochemical devulcanization by Zhang et al. [50]. In that study, 100 parts of GTR were mixed with 10 parts of aromatic oil with different contents of thiobisphenols of up to 3 g by a blender at room temperature. Subsequently, the devulcanization process was carried out using an internal mixer at 45 rpm between 180 and 200 °C for 10 min [50].

Ghorai et al. [8,51] proposed using bis(3-triethoxysilyl propyl) tetrasulfide (TESPT) for chemical devulcanization. Dubkov et al. [52] used N_2_O in organic solvents.

Sabzekar et al. [53] deployed benzoyl peroxide (BPO) as a devulcanizing agent. In addition, N-cyclohexyl-benzothiazyl-sulphenamide (CBS), tetramethylthiuram disulfide (TMTD), 2-mercaptobenzothiazol (MBT), and N-tert-butyl-2-benzothiazyl-sulphonamide (TBBS) could be deployed successfully for the devulcanization of cured rubber. Amines are another class of devulcanizing agents according to Sutanto et al. [54], e.g., hexadecylamine (HDA) [35].

Mangili et al. have compared different devulcanization methods [55]. The scCO_2_ (with DD) and ultrasonic methods as bulk treatments involve a high amount of energy and chemicals; however, they are quite selective processes. On the other hand, the biological process (using e.g., the bacterium *G. desulfuricans 213E*) is limited to the surface and is highly selective toward sulfur; it requires a low amount of energy and chemicals [55]. However, this process does not have high yields [55].

To study devulcanization, researchers have used ground tires, or they have prepared fresh ground natural rubber (GNR). For instance, in [8], Ghorai et al. prepared GNR from vulcanized natural rubber through compounding NR (100 phr = per hundred resin) with ZnO: 5 phr, stearic acid: 2 phr, CBS: 1.2 phr, and sulfur: 1.8 phr in a two-roll mixing mill at a friction ratio 1:1.25. Then, the compounded NR was cured at 150 °C for 3.5 min, followed by aging at 70 °C for 96 h. The vulcanized and aged rubber sheets were ground in a two-roll mixing mill to obtain GNR.

To analyze the quality of devulcanization, energy-dispersive X-ray (EDX), Fourier transform infrared spectroscopy (FTIR), field emission scanning electron microscope (FESEM) and thermogravimetric analysis (TGA) were used by Saputra et al. [49].

In addition, solvent extraction and swelling, as well as attenuated total reflectance Fourier transform infrared (ATR-FTIR) spectroscopy, were applied by de Sousa at al. [28], alongside cross-link density, soluble fraction, and Mooney viscosity, and by using the Horikx diagram by Seghar et al. [18]. Mangili et al. used cross-link density, sol fraction, gel fraction, and sulfur content [46].

In order to reduce the processing costs of “full devulcanization”, the dynamic vulcanization of GTR/plastic blends was proposed. This is a cross-linking process between GTR and a plastic matrix initiated by sulfur [56,57] or peroxides [58,59,60] during melt blending. According to Jiang et al., the resulting cross-linking will improve interfacial adhesion [11].

Another approach is to limit devulcanization to the surface of ground rubber tire powder. Thereby, particles can be reactivated to incorporate them into a new polymer matrix. This was investigated for PE by Jiang et al. [11] to prepare ground tire rubber/high-density polyethylene (GTR/HDPE) blends.

**Figure 6:**
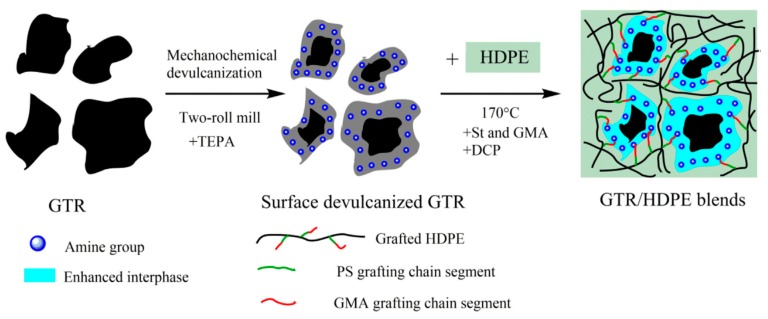
Schematic illustration of the preparation of ground truck-tire rubber/high-density polyethylene (GTR/HDPE) blend by combining surface devulcanization and in situ grafting technology (styrene (St), glycidyl methacrylate (GMA), and dicumyl peroxide (DCP) were used). Reproduced with permission from [15].

Surface devulcanization was achieved using intense shear and tetraethylenepentamine (TEPA), and then amine groups were grafted to the surface of devulcanized GTR by Jiang et al. [11]. In that paper, GTR was masticated in a two-roll mill with minimum roller distance (for maximum mechanical shear forces) 20 times. Then, 5 wt % TEPA as the chemically devulcanizing agent was added into the GTR, and the mixture was kneaded on the two-roll mill for 10 times to obtain surface-devulcanized GTR. The process was followed by in situ grafting; see Figure 6 below.

An innovative devulcanization/rubber reclaiming method is presented in [13] by Dobrotă and Dobrotă (ultrasonic activation).

### 2.4. Revulcanization

For revulcanizing the devulcanized rubber, the following recipe has been suggested.

On 100 parts of devulcanized rubber, 2 parts of stearic acid, 4 parts of ZnO, 1.5 parts of CBS (N-cyclohexyl-2-benzothiazole sulfonamide), and 1.5 parts of S were used by De et al. [16].

In [50], 100 parts of devulcanized rubber were mixed with 2.5 g of zinc oxide, 0.3 g of stearic acid, 0.8 g of accelerator NS (N-tert-butyl-2-benzothiazylsulfonamide), and 1.2 g of sulfur. Curing was done at 145 °C and 15 MPa.

### 2.5. Potential Advantages of Rubber Recycling

Reclaiming rubber from end-of-life products such as tires bears several advantages, as elaborated on by the pertinent literature [27]:

* Conservation of natural resources (less natural rubber is needed)
* Conservation of energy (less transportation, less energy in manufacturing)
* Avoidance of uncontrolled or high-emission end-of-life scenarios such as dumping or burning.
* Cost savings for goods producers, since the devulcanized material is cheaper than its replacement, natural rubber.

## 3. Results

Sabzekar et al. succeeded in adding 40% of reclaimed (devulcanized) rubber to natural rubber without a significant decrease in the mechanical properties [53].

It was found by Seghar et al. that up to 65 wt % of virgin natural rubber (NR) can be replaced by rubber recycled with devulcanized material [18].

Several studies confirm that devulcanized rubber can be reprocessed into rubber products such as tires without adverse effects.

Some of the studies have used laboratory equipment, such as roller mixers, while others have utilized industrial equipment such as co-rotating twin-screw extruders.

There are commercial offerings available, e.g., by Tyromer [61], Phenix [62], and Levgum [63]. Examples of two early patents are GB297817 (Firestone, 1935, Improvements in or relating to process of disintegration and devulcanization of rubber scrap) and GB2350839 (Goodyear, 2000, Surface devulcanization of cured rubber crumb).

## 4. Conclusions

**Figure 7:**
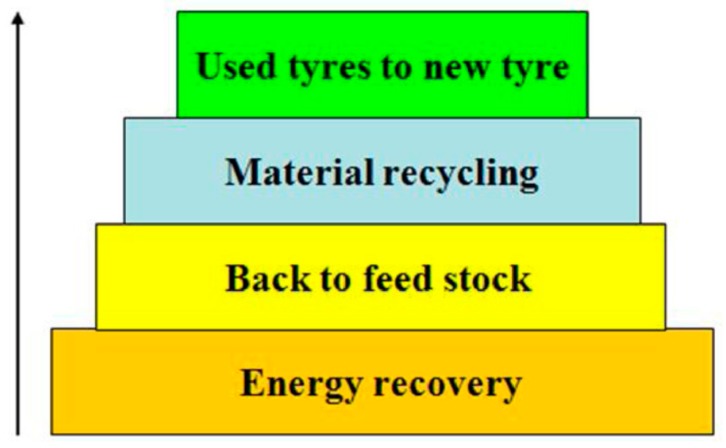
Proper waste management hierarchy. Reproduced with permission from [21].

The literature bears a wealth of information on rubber devulcanization, which can be achieved by thermal, thermochemical, mechanical, and biological means. The process as such has a good environmental performance, since virgin materials and energy are conserved. In addition, it can bring about significant cost savings. The general recycling hierarchy, which also applied to tires, is summarized in the following Figure 7.

Reuse is better than recycling, and a material recycling path is to be preferred over feedstock recycling due to the lower energy requirements. Energy recovery should be the last step of a cascaded use model. Landfilling in general should be avoided. Although carbon is being sequestered, the burying of organic, reactive materials bears risks, and waste tire dump fires have been reported previously, see e.g., Escobar-Arnanz et al. [22,64].

The same properties for rubber that has been devulcanized and revulcanized as for virgin material were reported by Ghosh et al. [65].

Apart from addressing the recycling of large volume rubber product streams such as tires, solutions need to be found to:

(a) make raw material manufacturing (i.e., latex/natural rubber) more sustainable
(b) make attrition to microplastics particles from tires less harmful, i.e., biodegradable. This might be achieved through suitable bioplastics materials.

Natural rubber today is mainly produced from the latex of the rubber tree or others. The rubber tree is grown in tropical areas, where plantations have often been established on previous rainforest land. Due to its nature to partially crystalize, natural rubber is harder than synthetic rubber, and it will give a longer lifetime to tires. This is also the reason while truck tires, which can run for well over 100,000 km [66], contain a larger fraction of natural rubber than do passenger car tires. Tire collection needs to be improved, and less environmentally friendly end-of-life options should be discontinued. There is a very strong, scientifically rooted interest in the feedstock recycling of rubber. On the one hand, this route provides a meaningful end-of-life exit for waste tires, and on the other hand, it conserves resources by reducing fresh natural and synthetic rubber demand. The circular economy concept [67] is to be extended to elastomers, in which tires will play a crucial role.

Other approaches to make tires more sustainable can be found in the use of alternative raw materials. For instance, Midhun et al. have suggested replacing carbon black (CB) by rice husk derived nanocellulose (RHNC) [68]; see also Fan et al. [69] for a carbon black outlook. Jiang [70] suggested using waste lignin to obtain a CB replacement material. Other novel fillers under discussion are functionalized starch (Li et al. [71]) and carbon nanotubes (Gumede et al. [72]).

## 5. Summary

Feedstock recycling can be considered the ultimate goal for polymers in that the original monomers are recovered. It seems feasible for some pure polymers. For complex product mixtures such as tires, feedstock recycling back to isoprene and the other constituents seems not feasible today. However, a process to reverse vulcanization, and hence make the elastomeric material meltable and processable again, is devulcanization. Devulcanization offers a route to recycling end-of-life tires back into high value-added products, so that virgin natural and synthetic rubber can be partly replaced and saved with economical and environmental benefits. 

This review article has provided an update on the state-of-the-art in rubber devulcanization, as a promising alternative to tire landfilling, grinding to powder and incineration in cement plants. Previous reviews are e.g., [73] by Manzano-Agugliaro et al., [74] by A. I. Isaye, [75] by Bockstal et al. [21], Forrest [15], De, Isayev, and Khait [16], Karger-Kocsis et al. [76], Simon et al. [77], and Garcia et al. [78].

Another area in need of more technology development is the natural rubber feedstock base. Today, it is dominated by latex from rubber trees. Alternative isoprene sources such as fig tree milk offer the potential to be more sustainable in terms of land usage, transportation, and cultivation requirements. Medium chain-length polyhydroxyalkanoates (mcl PHA) are biodegradable, and they can either be made from carbohydrates or through photoautotrophic microorganisms using CO_2_ as the sole carbon source. This offers the potential for biodegradable tires, where attrition would be significantly less harmful due to is shortened lifetime. In addition, microbial production would not require arable land and avoid competition with feed—and food—production, which is an issue often raised against biofuels and bioplastics. It is expected that the world fleet of cars will continue to grow, and that tires will be needed in future in large quantities. Therefore, sustainable end-of-life options are necessary, and more sustainable raw materials need to be sought. The circular economy concept needs to be extended to elastomers such as rubber and products made out of rubber. The devulcanization technology is a promising route with a realistic potential for large-scale implementation in the near future. Therefore, governments and the EU must introduce new laws for the circular economy and support companies to develop even more efficient recycling technologies.

## Figures and Tables

**Table 1:**
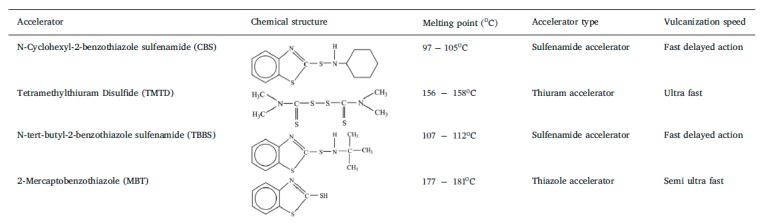
Chemical structure and physical characteristics of various accelerators. Reproduced with permission from [8].

**Table 2:**
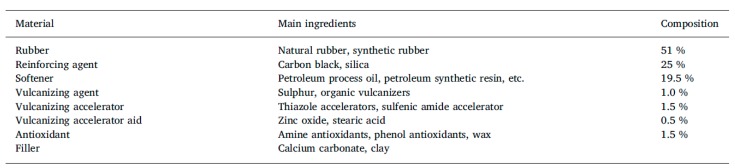
Tire rubber composition [22].
